# Evaluation of salt intake estimated from 24-h urinary sodium excretion in medical professionals in Darkhan-Uul Province, Mongolia: a cross-sectional study

**DOI:** 10.1038/s41598-023-37631-x

**Published:** 2023-06-28

**Authors:** Naoko Hikita, Enkhtungalag Batsaikhan, Satoshi Sasaki, Megumi Haruna, Ariunaa Yura, Otgontogoo Oidovsuren

**Affiliations:** 1 Department of Health Sciences, Graduate School of Medical Sciences, Kyushu University, 3-1-1 Maidashi, Higashi-Ku, Fukuoka, 812-8582 Japan; 2 Department of Midwifery and Women’s Health, Division of Health Sciences and Nursing, Graduate School of Medicine, The University of Tokyo, 7-3-1 Hongo, Bunkyo-Ku, Tokyo, 113-0033 Japan; 3 Department of Nutrition Research of the National Center for Public Health, Peace Avenue -17, Ulaanbaatar, 210349 Mongolia; 4 Department of Social and Preventive Epidemiology, School of Public Health, Graduate School of Medicine, The University of Tokyo, 7-3-1 Hongo, Bunkyo-Ku, Tokyo, 113-0033 Japan; 5 Darkhan-Uul General Hospital, 13-Bag, Darkhan Soum, Darkhan-Uul, Mongolia; 6 Darkhan-Uul Medical School, Mongolian National University of Medical Sciences, P.O. Box 903, Darkhan-Uul, Mongolia

**Keywords:** Health care, Risk factors

## Abstract

This cross-sectional study aimed to estimate the daily salt intake of medical professionals working in public health facilities in Darkhan-Uul Province, Mongolia. We conducted a multiple logistic regression analysis to identify factors associated with their consumption of salt exceeding the recommended daily salt intake (≥ 5 g/day). A self-administered questionnaire and 24-h urine samples were used to obtain data on the participants’ salt intake. Of 338 participants, 159 completed the 24-h urine collection. The mean sodium excretion into urine was 122.3 mmol/day, which was equivalent to a mean salt intake of 7.7 g/day when the urinary excretion rate was considered as 93%. Body mass index was positively correlated with excess salt intake (adjusted odds ratio [AOR]: 1.27; 95% confidence interval [CI] 1.10–1.46), while age was negatively correlated with excess salt intake (AOR: 0.95; 95% CI 0.91–1.00). Participants who consumed ≥ 2 cups of salted *suutei tsai* (Mongolian milk tea) daily had a higher risk of consuming ≥ 5 g/day of salt than those who consumed ≤ 1 cup/day. The average estimated salt intake of the participants was higher than the recommended value. Medical professionals should be aware of factors associated with excessive salt consumption and make appropriate adjustments to minimize it.

## Introduction

Excess dietary sodium intake is associated with numerous adverse health effects, including hypertension^1^, cardiovascular diseases (i.e. ischemic heart disease), and stroke^2^. Moreover, consumption of a low-sodium diet reduces hypertension and confers other health benefits^3–5^. The World Health Organization (WHO) recommends a sodium intake of < 2 g/day (salt intake [NaCl] < 5 g/day) to lower systolic and diastolic blood pressures and associated risk of developing cardiovascular diseases^6,7^.

In Mongolia, cardiovascular diseases are the leading cause of death in adults^8,9^, and several health measures have been adopted by the Ministry of Health to address this issue. This includes promotion of physical exercise, weight control, tobacco smoking cessation, consumption of vegetables and fruits, and reduction of alcohol and salt intake^10^. Another study conducted more than 20 years ago reported that the average amounts of salt intake, as measured using spot urine samples in pregnant women, non-pregnant women, and men, were 15.6 g, 12.6 g, and 14.6 g, respectively^11^. In addition, another report showed that the average daily salt intake in the Mongolian population was 11.1 g^12^, more than twice the daily limit recommended by the WHO. However, details of salt intake evaluation methodology were not described in this report; thus, there have hitherto been no studies on Mongolian adults that provide a detailed description of the 24-h urine salt concentration—a gold standard for measuring salt intake^13^. To reduce salt intake, it is necessary to accurately ascertain the current daily intake among Mongolian adults. Moreover, they should be provided with health guidance on the benefits of salt reduction by medical professionals, as a primary source of health education^14^. Thus, it is of public health importance that medical professionals educate their patients on the benefits of salt reduction and encourage them to consume < 5 g of salt per day, as recommended by the WHO^6,7^.

A previous study that compared sodium excretion among dietitians and non-dietitians reported no significant difference between the two groups^15^. These results indicated that there was no association between nutritional knowledge and better dietary behavior with sodium excretion. Medical professionals are responsible for educating the Mongolian community regarding the importance of salt reduction; nevertheless, based on previous findings, medical professionals do not seem to adhere to the recommended daily salt intake. Thus, it is necessary to know their current daily salt intake levels and the associated factors. This will help develop effective health policies aimed at reducing the salt intake of the Mongolian population.

This study aimed to quantify salt intake among medical professionals in Mongolia and identify the risk factors associated with their excess daily salt intake.

## Materials and methods

### Study design and population

This cross-sectional study was conducted from September to October 2019, in Darkhan-Uul Province, Mongolia. Eight trained research assistants recruited the participants from 10 public health facilities, including the Health Department, one general hospital, three soum (village) hospitals, and five health centers. Eligible participants were medical doctors, associate medical doctors, midwives, nurses, and public health nurses working in public health facilities in Darkhan-Uul Province. Approval was obtained from the Director of the Health Department to conduct the study. The heads of the health facilities were informed regarding the schedule and objectives of the study in advance, and their consent was obtained prior to study initiation.

### Data collection

Trained research assistants visited each health facility and recruited medical professionals for our current study. A self-administered questionnaire was used to collect the participants’ information, namely their age, sex, occupation, knowledge of the recommended salt intake, use of oral antihypertensive medicine, and practices related to salt intake. Awareness of the recommended salt intake was evaluated based on whether they could mention 5 g/day as the recommended amount or not. To examine whether the participants had diseases that could affect their salt intake, they were asked the following questions: “Did you receive antihypertensive medicine during the last 2 weeks?” (yes/no); “Did you use diuretics during the last 2 weeks?” (yes/no); and “Have you ever been diagnosed with renal disease by a physician?” (yes/no). Additionally, questions regarding daily salt intake practices derived from the STEPS survey^10^ or previous studies^16,17^ were as follows: (1) “Do you use iodized salt?” (yes/no); (2) “Do you buy a low salt/sodium alternative?” (always, sometimes, or never); (3) “How often do you add salt or salted sauces to your food at the table?” (always, often, sometimes, rarely, or never); (4) “How much salt or salted sauces do you use when eating?” (a little, moderate, or a lot); (5) “How often do you add salt or salted sauces to your food while cooking or preparing meals?” (always, often, sometimes, rarely, or never); (6) “How often do you eat processed foods high in salt (i.e., ham, salami, cheese, pickles, bread, ramen)?” (twice daily or more, once daily, once every 2–3 days, or once every 4–6 days); (7) “Do you read the information concerning salt or sodium levels on food labels?” (always, sometimes, or never); (8) “Do you use spices other than salt when cooking?” (always, sometimes, or never); (9) “On average, how many cups of salted *suutei tsai* (Mongolian milk tea) do you drink daily?” (2 cups/day or more, 1 cup/day or less); and (10) “On average, how many times per day do you eat soup dishes, such as noodle soup?” (2 times/day or more, approximately once per day, once per 2–3 days or less). A previous study reported that a higher percentage of noodle soup consumption corresponds to a higher salt intake^17^. Thus, we also asked the participants the following question: “How much soup (liquid) do you consume when eating soup dishes?” (approximately 20% or less, approximately 40–60%, or approximately 80% or more).

We also obtained anthropometric measurements (body height, weight, and blood pressure). We used a digital sphygmomanometer (ES-H56, Terumo Corp., Tokyo, Japan), a digital body composition weighing scale (BC-758, Tanita Corp., Tokyo, Japan), and a height meter to measure the blood pressure, body weight, and body height, respectively. Blood pressure was measured by trained research assistants after obtaining completed self-administered questionnaires. They selected one side of the arm, and measurements were performed twice. Average systolic and diastolic blood pressure values were calculated, and individual body weight and height measurements were used to calculate the body mass index (BMI; weight [kg]/height [m]^2^).

### 24-h urine collection

Prior to the initiation of the study, participants were asked to prepare at least two empty plastic bottles ≥ 1 L by washing and drying them well. Trained research assistants explained the collection procedure of the 24-h urine samples to each participant using an explanatory leaflet. Subsequently, the participants were asked to empty their bladders by urinating into the toilet; this was considered the starting time of the 24-h urine collection. The starting time was documented on the recording paper, and the participants were asked to collect all the urine they voided from that time point till the same time the next day. Participants were provided with eight 500-mL disposable paper cups for urine collection and a plastic bag to contain the bottle. Urine was to be collected in the paper cups and poured into the bottle. The paper cups were to be disposed of after use, and the participants were asked to use a new paper cup each time they urinated. They were asked to record the urination time and estimate the urine volumes on the recording paper when they forgot to collect urine. The urine bottles were stored at room temperature. After the 24-h urine collection was completed, the research assistants collected the urine samples from the participants.

### Urinary data analysis

To measure the volume of the collected urine sample, research assistants used a measuring cup before separating into samples of approximately 7 mL each, storing them in a plastic tube, and keeping them frozen at – 20 °C until analysis. The remaining urine was discarded. The frozen urine samples were transferred to GYALS Medical Center, in Ulaanbaatar, where the sodium and potassium levels were analyzed using the electrode method. The creatinine level was analyzed using a creatinine kit (Sarcosine Oxidase Method; Shenzhen Mindray Bio-Medical Electronics Co. Ltd., Nanshan, Shenzhen, China).

With the constant rate of urinary creatinine excretion, 24-h creatinine excretion was used as a standard to determine when the urine collections were complete^18,19^. The completeness of 24-h urine collection was determined using the method developed by Joossens et al.^20^ A urinary creatinine level of ([mmol/day] × 113/(21 × body weight [kg]) < 0.6 was considered to indicate incomplete 24-h urine collection in women, compared to that of ([mmol/day] × 113/(24 × body weight [kg]) < 0.6 in men.

Adjusted urine volume was calculated as the sum of collected urine volume and estimated voided (uncollected) urine volume. The reported collection time [h] was also calculated as the product of adjusted urine volume and (24-h/reported collection time [h]). Sodium excretion was calculated using the following conversion: 1 mmol of sodium is equivalent to 58.5 mg of NaCl. Daily salt intake was calculated by estimating that approximately 93% of sodium was excreted into the urine^21^.

### Statistical analysis

Descriptive statistics were used to determine the participants’ characteristics and estimated amounts of salt intake. Categorical data are presented as n (%) and continuous variables as means ± standard deviations. We analyzed categorical data with the chi-square test and continuous variables with Student’s t-test, to compare the values between the groups with complete and incomplete urine collections and between the groups with estimated daily salt intake of < 5 g/day and ≥ 5 g/day, respectively. Participant characteristics (age, sex, and BMI) and variables showing significant differences after bivariate analysis (p < 0.1) were selected as independent variables; subsequently, multiple logistic regression analysis (forced entry methods) was performed to investigate the risk factors of daily salt intake of < 5 g/day. Multicollinearity was evaluated using Spearman’s rank correlation coefficient or Cramer’s V; when the correlation coefficient was > 0.4, one of the variables was removed from the multiple logistic regression analysis. All data were analyzed using IBM SPSS Statistics 25.0 for Windows (IBM Corp., Armonk, NY, USA), and all two-tailed p-values < 0.05 (two tails) were considered statistically significant.

### Ethical considerations

This study was approved by the Research Ethics Committee of the Graduate School of Medicine, the University of Tokyo, Japan (2019025NI-[1]) and the Ethical Review Board of Ach Medical University in Mongolia (No. 19/02/10). Informed consent was obtained from all participants involved in the study. This study’s protocol complied with the principles of the Declaration of Helsinki.

## Results

Of the 538 medical professionals (58 men and 480 women) working in public medical facilities in Darkhan-Uul Province, 352 agreed to participate in this study. We further excluded 14 medical professionals because they did not submit their 24-h urine samples despite having completed the questionnaire forms. The final study population consisted of 338 (62.8%) medical professionals.

### Characteristics of the study participants who completed/did not complete the 24-h urine collection

Table 1 shows the characteristics of the participants with and without complete 24-h urine collection. The mean age of the participants was 40.3 ± 11.1 years, and 320 (94.7%) of them were women. Regarding occupation, nurses outnumbered the other medical professionals (176; 52.1%), followed by medical doctors (91; 26.9%), associate medical doctors (38; 11.2%), midwives (25; 7.4%), and public health nurses (8; 2.4%). The mean BMI was 26.6 ± 4.4 kg/m^2^, and the mean systolic and diastolic blood pressures were 115.0 ± 15.0 mmHg and 73.2 ± 10.9 mmHg, respectively.

**Table 1:** Characteristics of participants who completed and did not complete 24-h urine collection (n = 338).

	All		Incomplete^b^		Complete		*p*^d^
	(n = 338)		(n = 179; 53.0%)		(n = 159; 47.0%)		
	Mean ± SD or n (%)		Mean ± SD or n (%)		Mean ± SD or n (%)		
Age (years)	40.3 ± 11.1		41.2 ± 11.4		39.2 ± 10.8		0.089
Age group (years)							0.499^e^
20–29	81	(24.0)	41	(50.6)	40	(49.4)	
30–39	84	(24.8)	40	(47.6)	44	(52.4)	
40–49	79	(23.4)	43	(54.4)	36	(45.6)	
50–59	94	(27.8)	55	(58.5)	39	(41.5)	
Sex							0.086^e^
Male	18	(5.3)	6	(33.3)	12	(66.7)	
Female	320	(94.7)	173	(54.1)	147	(45.9)	
Occupation							0.729^e^
Medical doctor	91	(26.9)	47	(51.6)	44	(48.4)	
Associate medical doctor	38	(11.2)	23	(60.5)	15	(39.5)	
Midwife	25	(7.4)	11	(44.0)	14	(56.0)	
Nurse	176	(52.1)	93	(52.8)	83	(47.2)	
Public health nurse	8	(2.4)	5	(62.5)	3	(37.5)	
Body height (cm) ^a^	159.2 ± 6.5		158.7 ± 5.9^a^		159.8 ± 7.1		0.122
Body weight (kg)	67.6 ± 12.2		70.0 ± 12.5		64.9 ± 11.3		< 0.001
Body mass index (BMI) (kg/m^2^) ^a^	26.6 ± 4.4		27.7 ± 4.4^a^		25.4 ± 4.0		< 0.001
BMI category^a^							< 0.001^e^
BMI < 18.5	4	(1.2)	1	(25.0)	3	(75.0)	
18.5 ≤ BMI < 25.0	123	(36.6)	48	(39.0)	75	(61.0)	
25 ≤ BMI < 30.0	144	(42.9)	81	(56.3)	63	(43.7)	
30.0 ≤ BMI	65	(19.3)	47	(72.3)	18	(27.7)	
Systolic blood pressure	115.0 ± 15.0^a^		116.5 ± 16.7^a^		113.3 ± 12.7		0.042
Diastolic blood pressure	73.2 ± 10.9^a^		74.1 ± 11.7^a^		72.2 ± 9.8		0.098
Adjusted total urine volume (ml/day)	1429.9 ± 615.7		1326.6 ± 618.7		1546.1 ± 592.9		0.001
Total Na excretion (mmol/day)	100.7 ± 51.9		81.6 ± 46.6		122.3 ± 49.0		< 0.001
Estimated daily NaCl intake (g/day)	–		–		7.7 ± 3.1^c^		

*SD* standard deviation.

^a^2 data were missing.

^b^For female individuals, (urinary creatinine [mmol/day] × 113) / (21 × body weight [kg]) of < 0.6 were considered incomplete urine collection. For male individuals, (urinary creatinine [mmol/day] × 113) / (24 × body weight [kg]) of < 0.6 were considered incomplete urine.

^c^Calculated that approximately 93% of Na is excreted in urine. 1 mmol Na = 58.5 mg NaCl (salt).

^d^Student t-test.

^e^Chi-squared test.

The number of participants with complete 24-h urine collection, as determined using the method described by Joossens et al.^20^, was 159 (47.0%). Bivariate analysis showed significant differences in body weight, BMI, systolic blood pressure, adjusted urine volume, and total sodium excretion between the two groups with and without complete urine collection. Those with complete 24-h urine collection had a mean total sodium excretion of 122.3 ± 49.0 mmol/day and a mean estimated daily salt intake of 7.7 ± 3.1 g/day when the urinary excretion rate, compared with the intake, was considered.

### Characteristics of participants with estimated daily salt intake of < 5 g/day and ≥ 5 g/day

Table 2 shows the characteristics of participants who completed 24-h urine collection and had an estimated daily salt intake of < 5 g/day and ≥ 5 g/day. There were 31 participants (19.5%) with estimated daily salt intake of < 5 g/day. Moreover, 83 (52.2%) participants were aware of the recommended salt intake. Bivariate analysis showed that the use of oral antihypertensive medicine tended to be different between the two groups (< 5 g/day and ≥ 5 g/day), as were age and BMI.

**Table 2:** Characteristics of participants with complete 24-h urine collection^a^ who had an estimated daily salt intake^b^ of < 5 g/day and ≥ 5 g/day (n = 159).

	Estimated daily NaCl (salt) intake (g/day)^b^				*p*^d^
	NaCl < 5 g/day		NaCl ≥ 5 g/day		
	(n = 31; 19.5%)		(n = 128; 80.5%)		
	Mean ± SD or n (%)		Mean ± SD or n (%)		
Age (years)	42.8 ± 11.8		38.3 ± 10.4		0.036
Age group (years)					0.093^e^
20–29	6	(15.0)	34	(85.0)	
30–39	6	(13.6)	38	(86.4)	
40–49	6	(16.7)	30	(83.3)	
50 +	13	(33.3)	26	(66.7)	
Sex					1.000^f^
Male	2	(16.7)	10	(83.3)	
Female	29	(19.7)	118	(80.3)	
Occupation					0.553^e^
Medical doctor	12	(27.3)	32	(72.7)	
Associate medical doctor	3	(20.0)	12	(80.0)	
Midwife	2	(14.3)	12	(85.7)	
Nurse	14	(16.9)	69	(83.1)	
Public health nurse	0	(0.0)	3	(100.0)	
Body height (cm)	159.1 ± 6.5		160.0 ± 7.2		0.541
Body weight (kg)	60.8 ± 8.5		65.9 ± 11.7		0.008
Body mass index (BMI) (kg/m^2^)	24.0 ± 2.5		25.7 ± 4.2		0.004
BMI category					0.013^e^
BMI < 18.5	0	(0.0)	3	(100.0)	
18.5 ≤ BMI < 25.0	22	(29.3)	53	(70.7)	
25 ≤ BMI < 30.0	9	(14.3)	54	(85.7)	
30.0 ≤ BMI	0	(0.0)	18	(100.0)	
Having knowledge about recommended salt intake					0.259^e^
Yes	19	(22.9)	64	(77.1)	
No	12	(15.8)	64	(84.2)	
Systolic blood pressure	114.0 ± 12.9		113.41 ± 12.6		0.731
Diastolic blood pressure	73.5 ± 10.6		71.8 ± 9.6		0.398
Use of oral antihypertensive medicine					0.083^f^
Yes	8	(34.8)	15	(65.2)	
No	23	(16.9)	113	(83.1)	
Use of oral diuretic^c^					0.355^f^
Yes	1	(50.0)	1	(50.0)	
No	30	(19.2)	126	(80.8)	
Having diagnosis of renal disease					0.263^e^
Yes	14	(24.1)	44	(75.9)	
No	17	(16.8)	84	(83.2)	

*SD* standard deviation.

^a^For female individuals, (urinary creatinine [mmol/day] × 113)/(21 × body weight [kg]) of < 0.6 were considered as incomplete urine collection. For male individuals, (urinary creatinine [mmol/day] × 113)/(24 × body weight [kg]) of < 0.6 were considered as incomplete urine.

^b^Calculated that approximately 93% of Na is excreted in urine. 1 mmol Na = 58.5 mg NaCl (salt).

^c^1 data was missing.

^d^Student t-test.

^e^Chi-squared test.

^f^Fisher’s exact test.

### Daily practices of the participants related to salt intake

Table 3 shows the estimated daily salt intake of the participants and their practices related to it. In total, 128 (81.0%) participants used iodized salt, 48 (30.4%) did not add salt or salted sauce to food at the table, 59 (37.1%) did not read information on salt or sodium levels on food labels, and 70 (44.3%) consumed ≥ 2 cups of salted *suutei tsai* daily. Bivariate analysis showed significant differences in the consumption of salted *suutei tsai* between the two groups with daily salt intake of < 5 g/day and ≥ 5 g/day.

**Table 3:** Daily practices of the participants related to salt intake (n = 159).

	Estimated daily NaCl (salt) intake (g/day)				*p*^a^
	NaCl < 5 g/day		NaCl ≥ 5 g/day		
	(n = 31; 19.5%)		(n = 128; 80.5%)		
	n	(%)	n	(%)	
Using iodized salt (n = 158)					0.233
Yes	22	(17.2)	106	(82.8)	
No	8	(26.7)	22	(73.3)	
Buying low salt/ sodium alternatives (n = 157)					0.115^b^
Always	0	(0.0)	7	(100.0)	
Sometimes	22	(25.0)	66	(75.0)	
Never	9	(14.5)	53	(85.5)	
Frequency of adding salt or salted sauces to food at the table (n = 158)					0.787^b^
Always or often	2	(15.4)	11	(84.6)	
Sometimes	12	(23.1)	40	(76.9)	
Rarely	7	(15.6)	38	(84.4)	
Never	10	(20.8)	38	(79.2)	
How much salt or salted sauces do you use when adding at the table? (n = 146)					0.743
A little	24	(18.6)	105	(81.4)	
Moderate	4	(23.5)	13	(76.5)	
How often do you add salt or salted sauces to your food while cooking or preparing meals? (n = 158)					0.194^b^
Always	2	(33.3)	4	(66.7)	
Often	0	(0.0)	11	(100.0)	
Sometimes	8	(14.0)	49	(86.0)	
Rarely	12	(25.0)	36	(75.0)	
Never	9	(25.0)	27	(75.0)	
How often do you eat processed foods high in salt (ham, salami, cheese, pickles, bread, ramen, etc.)? (n = 143)					0.640^b^
2 times / day or more	1	(7.1)	13	(92.9)	
Once a day	3	(17.6)	14	(82.4)	
Once per 2–3 days	7	(23.3)	23	(76.7)	
Once per 4–6 days	16	(19.5)	66	(80.5)	
Reading the information on salt or sodium on food labels					0.687^b^
Always	1	(10.0)	9	(90.0)	
Sometimes	19	(21.1)	71	(78.9)	
Never	11	(18.6)	48	(81.4)	
Do you use spices other than salt during cooking? (n = 158)					0.688^b^
Always	2	(25.0)	6	(75.0)	
Sometimes	18	(20.7)	69	(79.3)	
Never	10	(15.9)	53	(84.1)	
Amount of salted *suutei tsai* consumed (n = 158)					0.021^b^
2 cups/day or more	8	(11.4)	62	(88.6)	
1 cup/day or less	23	(26.1)	65	(73.9)	
Frequency of soup dishes intake					0.749^b^
2 times/day or more	5	(15.2)	28	(84.8)	
Approximately once a day	13	(21.7)	47	(78.3)	
Once per 2–3 days or less	13	(19.7)	53	(80.3)	
How much soup (liquid) do you consume when eating soup dishes?					0.388^b^
Approximately 20% or less	3	(23.1)	10	(76.9)	
Approximately 40–60%	9	(27.3)	24	(72.7)	
Approximately 80% or more	19	(16.8)	94	(83.2)	

^a^Fisher’s exact test.

^b^Chi-squared test.

### Factors associated with exceeding the recommended daily salt intake

Bivariate analysis revealed that using oral antihypertensive medicine and consuming salted *suutei tsai* was related to adherence to the recommended daily salt intake. Multicollinearity between these variables and the participants’ characteristics (age, sex, and BMI) was not observed; thus, all variables were included in the multiple logistic regression analysis.

Table 4 shows the factors associated with adherence to the recommended daily salt intake. Higher BMI was a risk factor for consuming ≥ 5 g/day of salt (adjusted odds ratio [AOR]: 1.27; 95% confidence interval [CI] 1.10–1.46), while age was negatively correlated with consuming ≥ 5 g/day of salt (AOR: 0.95; 95% CI 0.91–1.00). Compared with participants who consumed ≤ 1 cup/day of salted *suutei tsai*, those who consumed ≥ 2 cups/day had higher odds of exceeding 5 g/day of salt intake (AOR: 3.91; 95% CI 1.46–10.45).

**Table 4:** Factors related to exceeding recommended daily salt intake.

	Crude odds ratio	95% CI	*p*	Adjusted odds ratio	95% CI	*p*
Age (years)	0.96	(0.93–1.00)	0.038	0.95	(0.91–1.00)	0.031
Sex						
Male	Reference			Reference		
Female	0.81	(0.17–3.92)	0.797	1.45	(0.26–8.22)	0.673
Body mass index (kg/m^2^)	1.14	(1.01–1.27)	0.030	1.27	(1.10–1.46)	0.001
Use of oral antihypertensive medicine						
Yes	Reference			Reference		
No	2.62	(1.00–6.90)	0.051	3.12	(0.90–10.78)	0.072
Amount of salted *suutei tsai* consumed						
2 cups/day or more	2.74	(1.14–6.59)	0.024	3.91	(1.46–10.45)	0.007
1 cup/day or less	Reference			Reference		

Multiple logistic regression analysis adjusted for the variables in this table. CI: Confidence interval.

## Discussion

This study is the first to report daily salt intake estimated from 24-h urinary sodium excretion and risk factors related to excess daily salt intake among medical professionals working in public health facilities in Darkhan-Uul Province, Mongolia.

In this study, the mean sodium excretion into urine was 122.3 mmol/day among participants who had completed 24-h urine collection. It was equivalent to the mean salt intake of 7.7 g/day when the urinary excretion rate was 93%. Additionally, only 19.5% of participants had a salt intake of < 5 g/day, as recommended by the WHO. Our results indicated that although medical professionals are responsible for delivering accurate medical guidance to the community, they themselves failed to adhere to the recommended salt intake (< 5 g/day). More than half (52.2%) the medical professionals in our study were aware of the salt intake recommendations; nevertheless, we found no association between their knowledge of the recommended salt intake and their actual salt intake. This result supports a previous finding showing that salt-related knowledge was not associated with salt intake^22^. Medical professionals are better informed regarding the necessity of salt reduction than the general population; hence, their salt intake is expected to be lower than that of the general population. The obtained value in this study (7.7 g/day) was lower than that observed in a previous report (11.1 g/day)^12^; however, the method of salt intake evaluation was not described in that the previous study, warranting further studies to accurately determine the average salt intake in the general Mongolian population, since salt-related knowledge was not associated with salt intake.

Although 24-h urine collection is considered the gold standard for measuring salt intake^13^, our study was conducted during a period characterized by cool-to-mild temperatures; the average daytime and nighttime temperatures were 20 °C and 9.4 °C, respectively^23^, and average humidity was 60%^24^ in September 2019, potentially causing excretion of some sodium in sweat. Furthermore, participants were on duty while participating in this study; thus, they sweated more, and sodium excretion might be systematically underestimated because we calculated that approximately 93% of sodium is excreted into the urine^21^. The actual salt intake among medical professionals could be higher than that presented in this study. Furthermore, we collected a single 24-h urine sample, which may not reflect the usual (habitual) sodium intakes. A previous study highlighted that because of the day-to-day variation in an individual’s dietary intake, the distribution of sodium excretion using a single 24-h urine sample becomes wide^25,26^.

In line with previous studies^25,27,28^, our study showed that a higher BMI value is a risk factor for exceeding the recommended salt intake because such individuals are inclined to consume more foods or poorly nutritious foods high in calories and salt. The opposite causal relationship has also been shown, with high salt intake associated with an increased risk of obesity^29^. As this was a cross-sectional study, causal relationships were not clear. Therefore, further investigations are required to elucidate such relationships.

In this study, the estimated daily salt intake significantly decreased with age, which supported the findings of a previous study^30^. In the STEPS survey performed in Mongolia, it was reported that the sodium concentration in spot urine samples was the highest among participants aged 25–34 years and the lowest among those aged 45–55 years^10^. This may be attributed to the fact that older people generally consume lesser amounts of food than younger people. In fact, the sodium intake per 1000 kcal among older people exceeded that among younger people^30^.

Furthermore, participants who consumed ≥ 2 cups/day of salted *suutei tsai* had a higher risk of exceeding the recommended salt intake, compared with those who consumed ≤ 1 cup/day. This result is consistent with the finding of a previous study that the salt intake of participants who drank salty tea was higher than that of those who did not^16^.

### Strengths and limitations

Our study has several strengths. First, we investigated daily salt intake using 24-h urine collection, which is considered the gold standard, and we identified factors associated with exceeding the recommended salt intake among medical professionals working in public health facilities in Mongolia. As medical professionals have the responsibility for delivering health education to the community, it is important to evaluate their daily salt intake levels. Further, as medical professionals are likely to possess more knowledge regarding the benefits of reduction of salt consumption, it can be inferred that the sodium intake of the general population would be higher than that of medical professionals. Furthermore, this study targeted all medical professionals who worked in public health facilities in the province; thus, the results of this study should be considered during the formulation of policies regarding the reduction of salt consumption in Mongolia.

Nevertheless, this study has some limitations. First, the response rate was only 62.8% of all eligible participants. We were unable to include all medical professionals who qualified for the study as a few of them were on holidays, some worked night shifts, and others declined to participate. Furthermore, female medical professionals in our study accounted for 94.7% of all participants, while the overall proportion of female medical professionals in Darkhan-Uul Province was 89.2%. Because of the low proportion of male medical professionals in this study, our results may not reflect the actual conditions in the province. Second, the number of complete 24-h urine samples was low, with only 159 (47.0%) samples considered to be complete. The 24-h urine sample collection required time and effort, thus preventing some of the participants from completing the study. Although the completeness of 24-h urine collection was determined using the method developed by Joossens et al.^20^, some of the participants’ urine volumes could be lower than the true volume in this study, even among those with complete urine collection. This would affect our findings, and underestimation is possible. Although we asked the participants to record the estimated volumes of voided urine when they forgot to collect it, so that the uncollected urine volumes could be added during calculations, the rate of complete 24-h urine sample collection remained low. Furthermore, despite the positive correlation between the BMI and salt intake, there were significant differences in the BMI between participants who completed the 24-h urine collection and those who did not. This indicates that if individuals excluded from the analysis had been able to complete the 24-h urine collection, the estimated amount of salt intake might have been higher than the values reported in this study. Third, we collected only a single 24-h urine sample. However, even with a single 24-h urine collection, the mean 24-h urinary sodium excretion can provide a good estimate of the mean daily sodium intake of the group^20^; thus, these results could provide useful information. Fourth, some unmeasured confounding factors could have influenced urinary creatinine excretion, such as emotional stress, acute infection, fever and trauma, and renal insufficiency^31^, as reported in previous studies. Fifth, the questionnaire employed to evaluate the daily salt intake practices comprised original questions, and its validity and reliability were not examined. Therefore, in this study, only the consumption of salted *suutei tsai* was considered an indicator of salt intake. Furthermore, it is important to note that the assessment of participants’ knowledge regarding salt was based on a single question, which may not provide a comprehensive measure of their overall salt intake-related knowledge and awareness. Further research is required to address these limitations.

## Conclusions

The estimated daily salt intake among medical professionals in Mongolia was 7.7 ± 3.1 g/day when considering urinary excretion rate compared to intake. Although medical professionals assume the responsibility for providing health education to the community, most of them failed to follow the recommended salt intake guideline. Higher BMI, younger age, and consuming ≥ 2 cups of salted *suutei tsai* daily contributed to the participants exceeding the recommended salt intake in this study. All medical professionals and policymakers should be aware of factors associated with daily salt intake in order to reduce their daily salt intake. A similar study including participants from the general population of Mongolia should be performed in the future.

## Data Availability

The datasets used and/or analyzed during the current study are available from the corresponding author on reasonable request.
